# New Method of Treating Urethral Pains Following Gonorrhea

**Published:** 1849-08

**Authors:** 


*New Method of Treating Urethral Pains following Gonorrhea.*—M. Vidal (de Cassis) having frequently remarked that these pains were relieved by pressing the penis with the fingers, has been led to try compression for their treatment, and has found it useful, affording a perfect cure in many cases, and a marked alleviation in others. The operative procedure, says M. Vidal, is so simple that it is scarcely necessary to mention it. "The surgeon takes a long strip of diachylon plaster, one centimeter (two-fifths of an inch) in breadth, and rolls it around the penis in the same manner as a common bandage, beginning at the glans; or, still better, he may apply it more accurately by using a number of small strips of plaster, each of which shall only be sufficient to encircle the organ once, and the two extremities of each strip should be made to cross upon the urethra, for the purpose of insuring the firmness of the dressing. The principal point to be attended to is the degree of compression, which ought to be as firm as possible, without interfering with micturition, which would, of course, necessitate the removal of the dressings. The compression should be continued for a considerable period after the cessation of the pains, to prevent their return." M. Vidal cites two cases, from amongst great numbers which he has treated, in favor of this mode of practice.—*Monthly Retrospect*, from *L'Union Médicale.*

